# Evaluation of Efficacy of Three Different Commercially Available Kit for Chairside Cariogenic Bacteria Test – Caries Risk Test, Saliva-check Mutans and CariScreen

**DOI:** 10.7759/cureus.6504

**Published:** 2019-12-29

**Authors:** Vidya Babu, Sundeep Hegde, Sham Bhat, Sharan Sargod

**Affiliations:** 1 Pedodontics and Preventive Dentistry, Yenepoya Dental College, Mangalore, IND

**Keywords:** chairside kits, culture-based detection, caries risk test, cariscreen, salivacheck mutans

## Abstract

Background and objectives

The presence of mutans streptococci has been used in individual assessments of caries risk. In the modern era of dentistry, the chair side kits for assessing chair side cariogenic bacteria play a significant role. There is paucity of literature about the comparison of commercially available chair side caries risk tests. Hence this study was conducted to compare the efficacy of three commercially available chair side cariogenic bacteria tests.

Methodology

Twenty-five patients in the age group of 5-14 years were selected. The saliva samples of patients were collected and were taken for cariogenic bacteria tests using caries risk test (CRT) bacteria test kit and saliva check mutans kit (mutans rapid detection kit). The plaque samples were taken for CariScreen caries susceptibility testing meter. All the samples were compared with a gold standard, i.e., mitis salivarius-bacitracin (MSB) agar plate test.

Results

Results demonstrated that the specificity of CariScreen and caries risk test was 91.67 whereas it was 75.00 for saliva check mutans. The CariScreen produced the risk status of the patient in shortest time. However, all the chair side kits failed to show the exact colony count of bacteria.

Conclusion

The result of the current study proved that both CariScreen and caries risk test are highly efficient in assessing the caries risk of patients. However, the CariScreen is easy to perform and provides the result in shorter time.

## Introduction

Bacteria play a crucial role in the formation and prognosis of dental caries. Among the clinical risk factors, mutans streptococci play an important role [1]. Various studies suggest an inverse relationship between the prevalence of non-mutans streptococci and the mutans streptococci, and is also correlated with caries development [2]. The period of highest risk for caries incidence in permanent teeth was the first few years after tooth eruption [3]. A variety of test assays are commercially available for measuring the abundance of Streptococcus mutans in saliva. Of these, widely used are the culture-based detection assays. However, to overcome the disadvantage of short shelf life and low specificity chair side kits were introduced. The CariScreen caries susceptibility testing meter is a chair side caries risk assessment tool which utilizes adenosine triphosphate (ATP) bioluminescent liquid. The caries risk test (CRT) kit uses selective culture media for caries risk assessment. The saliva check mutans is a chair side kit that uses specific immunochromatography process. The literature is lacking about the comparison of commercially available chair side caries risk tests. Hence the purpose of this study is to evaluate the efficacy of three different commercially available kit for chair side cariogenic bacteria test - caries risk test, saliva-check mutans and CariScreen.

## Materials and methods

The study was conducted in Department of Pedodontics and Preventive dentistry, Yenepoya Dental College in collaboration with the Yenepoya Research Centre, Mangalore. Ethical clearance was obtained from the institutional ethical committee. Twenty-five patients in an age group of 5-14 years were selected. The children with moderate caries and above were included in the study after performing the DMFT index and/or dmft index. The children undergoing radiation therapy and antibiotic therapy were excluded from the study. The duration of the study was one year. Stimulated saliva samples were required for two kits (CRT bacteria test kit and saliva check mutans test) while plaque sample was required for CariScreen test. Stimulated saliva was collected after giving paraffin tablets and plaque samples were collected from teeth surfaces. All the samples were compared with a gold standard, i.e., mitis salivarius-bacitracin (MSB) agar plate test. The saliva samples were divided into three groups - group A, B and C. Group A underwent test using CRT bacteria test kit (VIVADENT), group B underwent test for saliva-check mutans (GC) and group C was taken for MSB agar plate test. The plaque samples were taken for CariScreen caries susceptibility testing meter which was group D.

**Figure 1:**
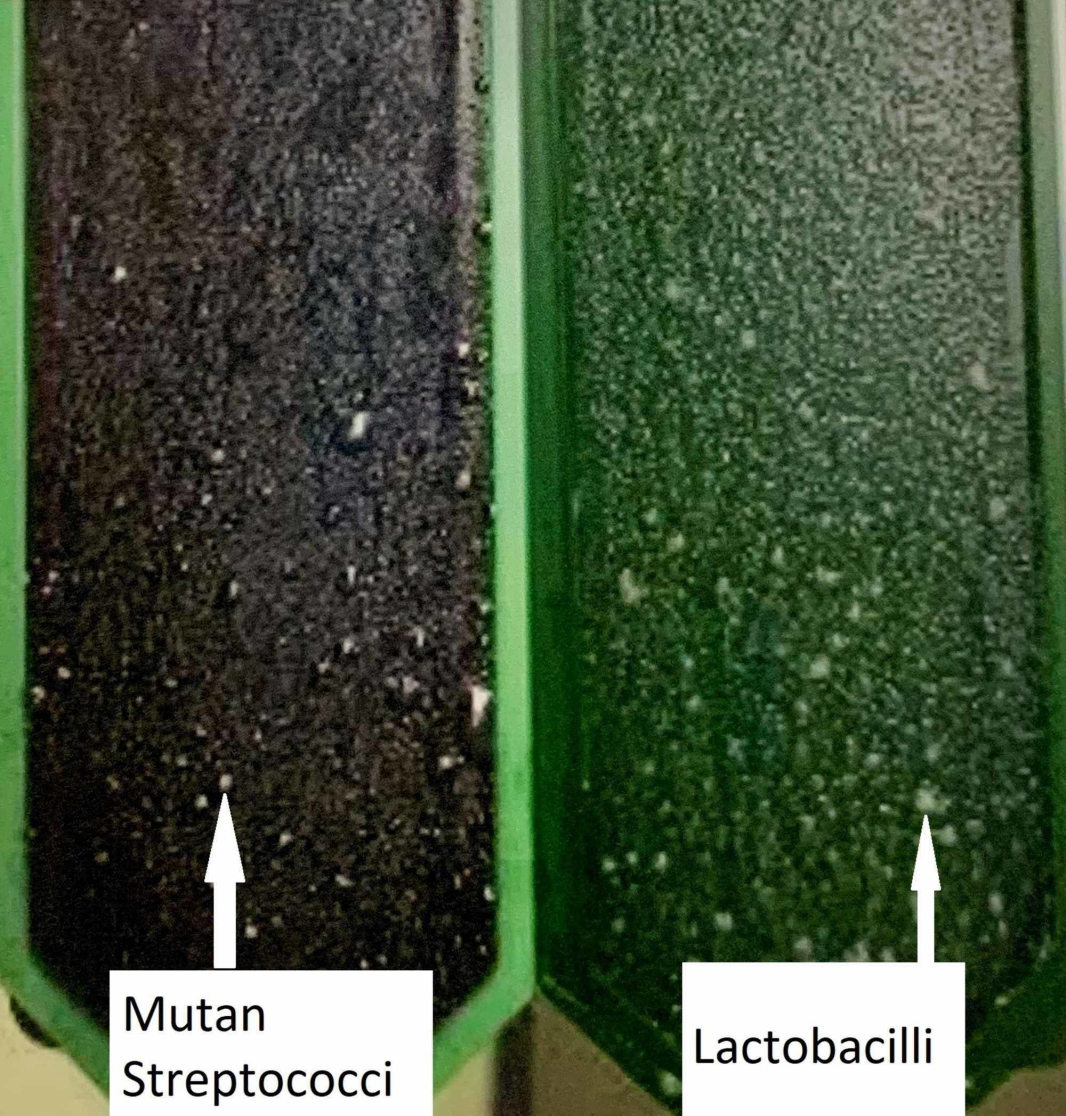
Mutan streptococci and lactobacilli colony on test vial after incubation

Group A - The collected stimulated saliva was spread on to the agar surface on the test vial. The test vial was then placed in an incubator at 37°C for 48 hours. After removal of the vial from the incubator, the density of the mutans streptococci and lactobacilli colonies were compared with the corresponding evaluation pictures in the enclosed model chart (Figure 1).

**Figure 2:**
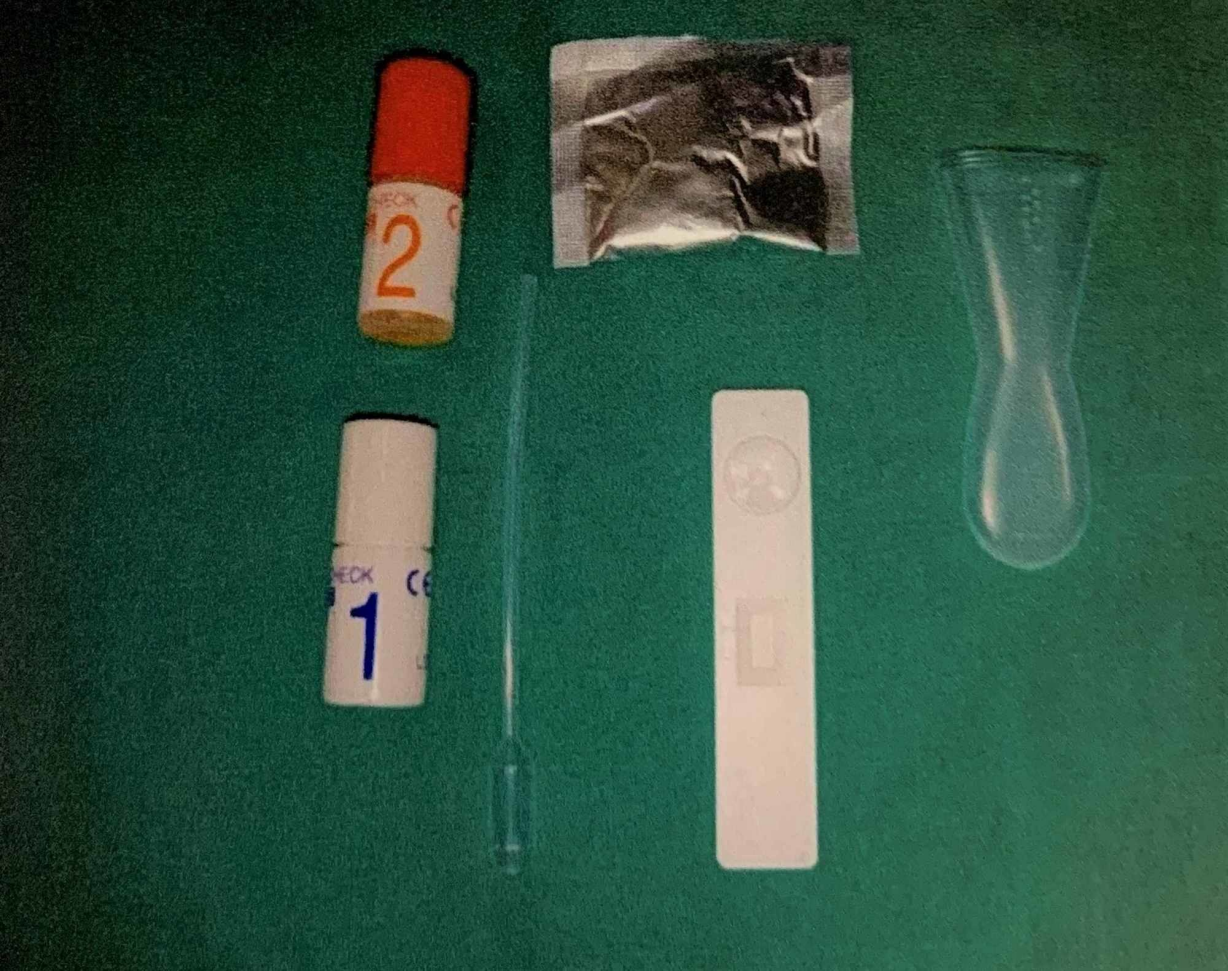
Contents of saliva check mutans

**Figure 3:**
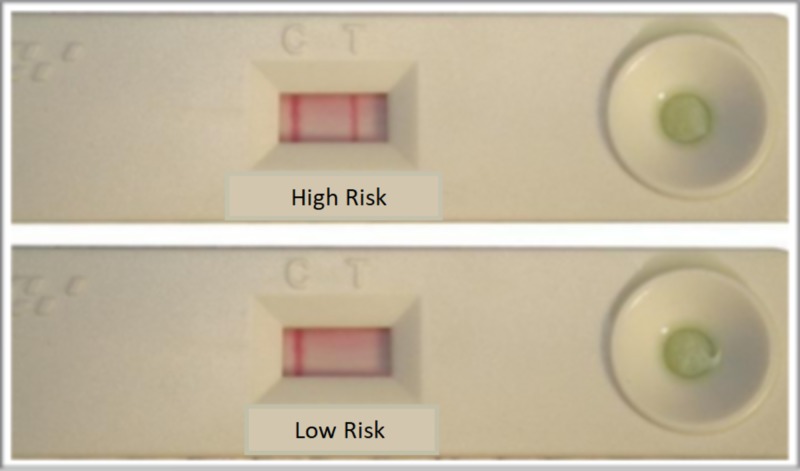
Result of saliva check mutans after 15 minutes

Group B - The collected stimulated saliva was mixed with the reagents given with the kit according to the manufacturer’s instruction following which the saliva was dispensed into the test device. The results were assessed after 15 minutes (Figures 2, 3).

Group C - Group C was considered as the standard method. The saliva collected were spread on to the mitis salivarius agar base plate. The colony count was done after 24 hours.

**Figure 4:**
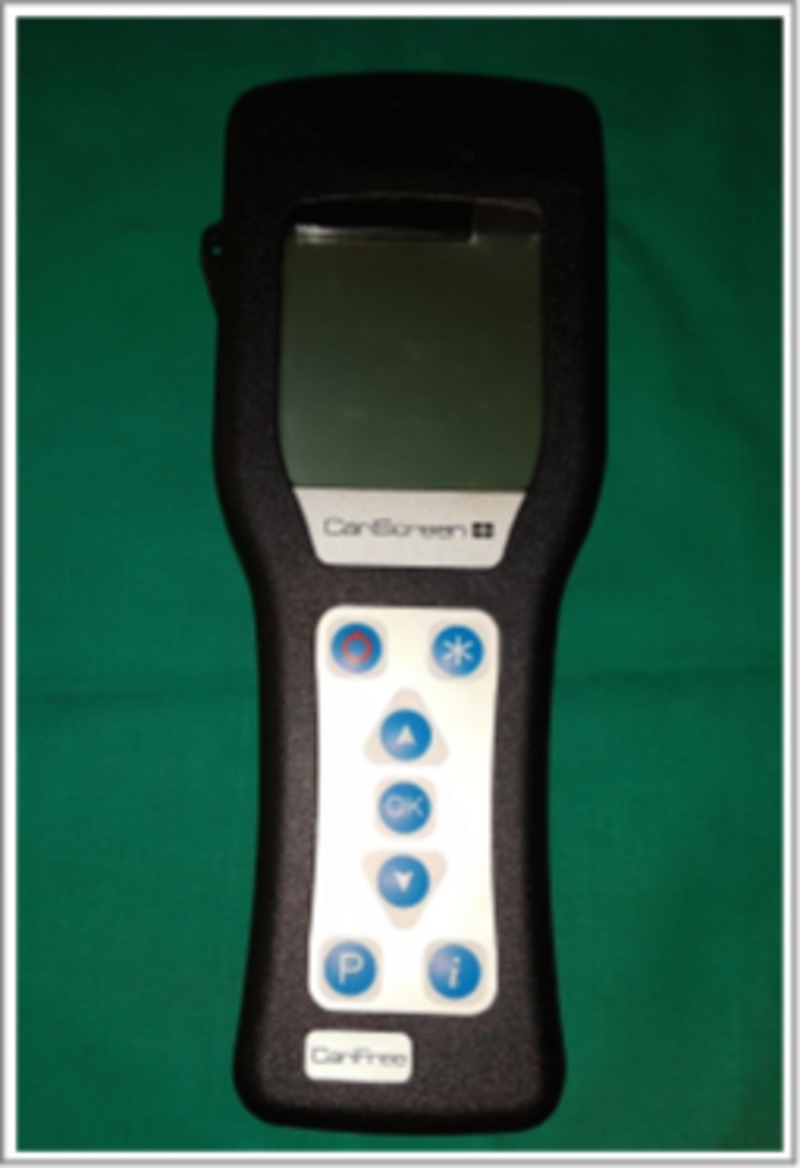
CariScreen caries susceptibility testing meter

Group D - Plaque samples were collected using a swab provided in the chair side kit. The swab was mixed with the ATP bioluminescent liquid present in the swab and the swab was then inserted into the device. The caries risk status was displayed on the device immediately (Figure 4).

The colony count of Group A, B and D was compared to that of the standard, i.e., group C. Sensitivity, specificity and positive and negative likelihood ratios are estimated by comparison with a known reference (gold standard) test. Results are presented as estimates of sensitivity and specificity with specified Clopper-Pearson (exact) confidence limits and point estimates of positive and negative likelihood ratios. Within and between groups comparison of sensitivity and specificity was calculated using McNemar's Chi-square test.

## Results

Out of 25 samples, 14 patients were categorized into high caries risk group and 11 were categorized into low caries risk group in group A. The specificity of caries risk test when calculated was 91.67 with a lower confidence level of 76.03 and upper confidence interval of 100.00 (Tables 1, 4).

**Table 1:** Cross tab between the conventional method and caries risk test.

		Conventional method	
		High N (%)	Low N (%)
Caries Risk Test	High	13 (100)	1 (8.3)
	Low	0	11 (91.7)

In group B out of 25 samples, 16 patients were in high carious risk group and nine patients were in low carious risk group. The specificity in this group is found to be 75.00 with a lower confidence interval of 50.50 and upper confidence interval of 99.50 (Tables 2, 4).

**Table 2:** Cross tab between the conventional method and saliva-check mutans

		Conventional method	
		High N (%)	Low N (%)
Saliva-check mutans	High	13 (100)	3 (25.0)
	Low	0	9 (75.0)

In group C, out of 25 samples, 13 patients were in high carious risk group and 12 were in low carious risk group. This group was considered as the standard and all the other groups were compared to this group.

In group D, out of 25 samples collected, 14 patients were in high caries risk group and 11 were in low caries risk group. The specificity of CariScreen is 91.67 with a lower confidence level of 76.03 and upper confidence interval of 100.00 (Tables 3, 4).

**Table 3:** Cross tab between the conventional method and CariScreen

		Conventional method	
		High N (%)	Low N (%)
CariScreen	High	13 (100)	1 (8.3)
	Low	0	11 (91.7)

**Table 4:** Diagnostic test to find the sensitivity, specificity, positive predictive value, negative predictive value and accuracy of the chair side kits. CI: Confidence interval; PPV: Positive predictive value; NPV: Negative predictive value.

	Saliva-check mutans (CI)	Caries risk test (CI)	CariScreen (CI)
Sensitivity	88.42	93.14	92.86
Specificity	75.00 (50.5-99.5)	91.67 (76.03-100)	91.67 (76.03)
PPV	81.25 (62.12-100)	92.86 (79.37-100)	92.86 (79.37-100)
NPV	100.00	100.00	91.67
Overall accuracy**	88.00 (75.26-100)	96.00 (88.32-100)	96.00 (88.32-100)
McNemars chi square	.00	.01	.50
P-value	1.0	1.0	.47

Within and between group comparison of sensitivity and specificity it did not show any significant difference among the groups. However, clinically while performing the tests, fastest result was obtained in group D > group B > group C > group A. The caries risk status was obtained in less than one minute in group D while group A had the longest waiting time to yield the result, i.e., 48 hours.

## Discussion

Despite the marked improvement in oral health, caries occurs in both developed and developing countries worldwide [4]. Dental caries is one of the most common chronic diseases affecting millions of people globally [5, 6]. Early childhood caries is a serious chronic oral health problem with an alarmingly high prevalence among children in both developed and developing countries, despite implementation of established caries management measures [7]. Dental caries activity tests have been widely used in the assessment, monitoring and motivation of patients with dental caries and still caries activity test is under the continuous challenge due to its multi-factorial nature [8]. However, caries is now considered to be a specific odontopathic infection, the principal causative organism being mutans streptococci [9]. The present study aims at comparing the efficacy of three commercially available kits for chair side cariogenic bacteria test and to assess the caries risk status. The chair side kits used in this study are CRT bacteria kit (Vivadent), CariScreen caries susceptibility testing meter and Saliva-check mutans kit (Streptococcus mutans rapid detection kit) (GC). While comparing the chair side kits with that of the conventional culture-based tests, CariScreen and CRT kits gave the highest specificity value. The CariScreen caries susceptibility testing meter is a chair side caries risk assessment tool which utilises ATP bioluminescent liquid. The material required for this test is the plaque sample which was easier to collect especially from younger patients. Also, the result will be displayed in about 15 seconds which saves the chair side time thus making it a reliable tool for assessing the caries risk of the patient with a specificity value of 91.67. Fazilat et al. have found that ATP measurements have a strong statistical association with bacterial number in plaque and saliva specimens, including numbers for oral streptococci, and may be used as a potential assessment tool for oral hygiene and caries risk in children [10].

The CRT and the saliva-check mutans kit required stimulated saliva which was unacceptable for children. Also, the waiting period of 48 hours for the caries risk assessment is a disadvantage of CRT. Though the saliva-check mutans is very easy to perform and the result can be obtained in 15 minutes, the specificity of this kit when compared to the standard is low (75.00). However, all the three chair side kits failed to give the exact colony count of bacteria present when compared to the standard, i.e., the conventional culture-based assay. This is in accordance with Ohmori et al. who have compared the performance of four commercial salivary test kits, Dentocult SM (Orion, Finland), CRT (Vivadent, Liechtenstein), CarioCheck SM (Sunstar, Japan) and saliva check SM (GC Japan) and concluded that none of the salivary test kits evaluated in his study was capable of detecting the accurate number of cariogenic bacteria or S. mutans [3]. Also, according to a study conducted by Hildebrandt and Bretz on comparison of culture media and chair side assays for enumerating mutans streptococci, they stated that chair side cultural tests displayed considerable disparity between tests in recovering bacteria from pure cultures [11]. Thus, chair side cariogenic bacteria kits is a practical way in identifying high microbiological caries-risk subjects. They are sensitive enough for screening purposes and for patient centered promotion of oral health. These kits also help the practitioners provide a baseline and assists in planning the treatment accordingly.

The drawback of the study is that the size of the sample is not adequate to draw an effective conclusion.

## Conclusions

The chair side cariogenic tests are efficient in early detection of caries which helps to formulate immediate treatment plan. The result of the current study proved that both CariScreen and caries risk test are highly efficient in assessing the caries risk of patients but the cost of CariScreen is a disadvantage. The CariScreen is easy to perform and provides the result in shorter time. However, a long-term study with higher sample size is required to substantiate our findings.
